# Responses of cuticular waxes of faba bean to light wavelengths and selection of candidate genes for cuticular wax biosynthesis

**DOI:** 10.1002/tpg2.20058

**Published:** 2020-10-30

**Authors:** Lei Huang, Qianlin Xiao, Xiao Zhao, Dengke Wang, Liangliang Wei, Xiaoting Li, Yating Liu, Zhibin He, Lin Kang, Yanjun Guo

**Affiliations:** ^1^ College of Agronomy and Biotechnology Southwest University Chongqing 400716 China

## Abstract

Cuticular waxes play important eco‐physiological roles in protecting plants against abiotic and biotic stresses and show high sensitivity to environmental changes. In order to clarify the responses of cuticular waxes on faba bean (*Vicia faba* L.) leaves to different light wavelengths, the phenotypic plasticity of cuticular waxes was analyzed when plants were subjected to white, red, yellow, blue, and purple light. Leaf samples from yellow, purple, and white lights were further analyzed, and candidate genes of wax biosynthesis were selected by RNA‐seq technology and transcriptome processing. Yellow light increased the total wax coverage and changed the crystal structure compared with leaves under white light. Light wavelengths changed the relative abundance of dominant primary alcohol from C_24_ under white, yellow, and red lights to C_26_ under blue and purple lights. In total, 100,194 unigenes were obtained, and 10 genes were annotated in wax biosynthesis pathway, including VLCFAs elongation (KCS1, KCS4, LACS2 and LACS9), acyl reduction pathway (FAR3 and WSD1), and decarboxylation pathway (CER1, CER3 and MAH1). qRT‐PCR analysis revealed that yellow and purple lights significantly influenced the expression levels of these genes. Yellow light also increased the water loss rate and decreased the photosynthesis rate. Light at different wavelengths particularly yellow light induced the changes of phenotypic plasticity of cuticular waxes, which thus altered the leaf eco‐physiological functions. The expression levels of genes related to wax biosynthesis were also altered by different light wavelengths, suggesting that light at different wavelengths may also be applied in selecting candidate genes involved in wax biosynthesis in other crops.

**Abbreviations**
ABC: ATP‐binding cassette
ACL: average chain length
ACP: acyl carrier protein
BSTFA: bis‐N,O‐trimethylsilyltrifluoroacetamide
Ci: intercellular CO_2_ concentrations
CPI: carbon preference index
DEGs: differentially expressed genes
ECR: trans‐2, 3‐enoyl‐CoA reductase
ER: endoplasmic reticulum
FAE: fatty acid elongases
FAR: fatty acyl‐CoA reductase
FDR: false discovery rate
FID: flame ionization detector
FPKM: fragments per kilobase of transcript per million mapped reads
GC: gas chromatography
Go: Gene Ontology
gs: stomatal conductance
HCD: 3‐hydroxacyl‐CoA dehydratase
KCR: 3‐ketoacyl‐CoA reductase
KCS: 3‐ketoacyl‐CoA synthase
KEGG: Kyoto Encyclopedia of Genes and Genomes
KOG/COG/eggNOG: Cluster of Orthologous Groups of proteins
LEDs: light emitting diodes
LSD: least significance difference
NR: NCBI non‐redundant protein sequences
ORF: open reading frames
Pfam: Protein family
PKS: polyketide synthase
Pn: photosynthesis rate
qRT‐PCR: quantitative real‐time reverse transcription‐PCR
RSEM: RNA‐Seq by Expectation Maximization
SEM: Scanning electron microscopy
Tr: transpiration rate
VLC: very long chain
VLCFAs: very long chain fatty acids.

## 1 INTRODUCTION

Cuticular wax is the outermost surface of plant organs and thus serves as the first barrier for plant defense (Jetter, Kunst, & Samuels, 2006; Ni, Guo, Zhao, & Guo, 2016). It helps restrict non‐stomatal water loss (González & Ayerbe, 2010; Kunst & Samuels, 2003), reduces water retention on the plant surface (Koch, Hartmann, Schreiber, Barthlott, & Neinhuis, 2006), and reduces harmful effects induced on plant tissues by enhanced ultraviolet radiation (Ni, Xia, & Li, 2014a). Responses of cuticular wax deposition to varying environments are related to plant adaptations (Yeats & Rose, 2013). Cuticular wax is also the first part of the process in which plants accept light. As an important energy source and signal, light plays a crucial role in plant survival and development and affects plant growth and architecture, photosynthesis rate, and crop yield (Avercheva et al., 2009; De Wit, Galvão, & Fankhauser, 2016). However, light sources vary by intensity and quality within modern agricultural production, such as the use of light emitting diodes (LEDs) with specific wavelength used in plant factory or plant growth chamber (Olle & Viršile, 2013). In nature, the depletion of the ozone layer induced by climate change also alters light wavelengths, particularly an increase in ultraviolet radiation (Moan, 2001; Schiedek, Sundelin, Readman, & Macdonald, 2007). Such changes in light wavelengths may influence cuticular wax deposition, and thus their eco‐physiological roles. Therefore, understanding the responses of plant cuticular waxes to changes in light will help us enhance crop adaptations to adverse climate changes and improve crop yield.

Cuticular waxes are composed of hydrophobic very long chain fatty acids (VLCFAs) and their derivatives, such as alkanes, primary and secondary alcohols, aldehydes, ketones, and alkyl esters (Guo & Jetter, 2017; Samuels, Kunst, & Jetter, 2008). The compositions of this wax vary greatly among plant species, organs, and growth stages (Jetter & Riederer, 2016; Zhao et al., 2019). Environmental changes also impose a direct effect on the composition and content of cuticular waxes. For example, drought induced an increased alkane content in wheat (Bi et al., 2017), cold stress induced an increase in alkanes and secondary alcohols of *Arabiodopsis thaliana* (Ni, Song, & Wang, 2014b), and the transfer of dark‐grown leaves to light resulted in 2.5‐fold greater wax yield in barley (Giese, 1975). Studies have also shown that high radiation could increase the wax deposition in brassica plants (Shepherd, Robertson, Griffiths, Birch, & Duncan, 1995) and increase the alkane accumulation in *Cucumis sativus* seedlings (Tevini & Steinmüller, 1987). The cuticular wax composition of *Nicotiana tabacum* leaves differed in their responses to light wavelengths (Chen, Jiang, Qiu, Jiang, & Pan, 2011). The dominant components of cuticular wax are usually attributed to the micromorphology formation of wax crystalliods. For example, platelets are dominated by primary alcohols, tubules always contain predominantly secondary alcohols or diketones, and threads are mostly consisted of flavonoids (Barthlott et al., 1998). Environment also imposes an effect on the ultrastructure of particular wax crystalliods (Baker, 1982). Enhanced UV‐B radiation would alter the crystalliods structure of epicuticular wax on *Arabidopsis* stems (Ni, Song, & Li, 2015a). Such changes in the physical and chemical properties of cuticular waxes induced by genetics and environments might alter its physiological and biochemical functions, and thus crop adaptations and resistance.

Wax biosynthesis begins with de novo C_16_ and C_18_ fatty acids biosynthesis in plastid of epidermis cells (Jenks, Tuttle, Eigenbrode, & Feldmann, 1995), then the C_16_ and C_18_ fatty acids are transferred to endoplasmic reticulum (ER), where they are catalyzed to VLCFAs by a membrane‐bound multi‐enzyme complex including 3‐ketoacyl‐CoA synthase (KCS), 3‐ketoacyl‐CoA reductase (KCR), 3‐hydroxacyl‐CoA dehydratase (HCD) and trans‐2, 3‐enoyl‐CoA reductase (ECR) (Kunst, Jetter, & Samuels, 2006). Currently, many genes and transcription factors involved in cuticular wax biosynthesis from *A. thaliana* and some crops have been identified (Lee & Suh, 2015). For example, 21 KCS genes were identified in *A. thaliana* (Dunn, Lynch, Michaelson, & Napier, 2004). KCS6/CER6/CUT1 is specifically involved in the elongation of C_24_ VLCFAs (Lee & Suh, 2013). ECR was shown to be identical to CER10, which is involved in all elongation cycles (Zheng, Rowland, & Kunst, 2005). CER4 and WSD1 encode enzymes involved in the acyl reduction pathway producing primary alcohols and alkyl esters (Li et al., 2008; Rowland et al., 2006). CER1 catalyzes the formation of alkanes, whereas MAH1 catalyzes alkane into secondary alcohols and ketones by the decarboxylation pathway (Bernard et al., 2012; Bourdenx et al., 2011; Greer et al., 2007). The identification of β‐diketones in *Hordeum vulgare* further supports another biosynthesis pathway existing in plants: polyketide synthase (PKS) (Hen‐Avivi et al., 2016). Some transcription factors involved in the regulation of cuticular wax biosynthesis have also been identified, including AP2/EREBP‐type (WIN1/SHN1, SHN2, SHN3) and MYB transcription factors (MYB30, MYB41, MYB96) (Li‐Beisson et al., 2013). This progress enables us to explore the molecular mechanisms of cuticular wax in responding to changing light wavelengths.

Genes involved in wax biosynthesis are also regulated by changing environments. For example, light is essential for CER6 transcription (Hooker, Millar, & Kunst, 2002). Photoperiod influenced the transcription of genes involved in the biosynthesis, transportation, and regulation of cuticular wax in *A. thaliana*, such as CER1, LTP7, LACS3, LTP6, LTP2 and ABCG19 (Go, Kim, Kim, & Suh, 2014). Some wax genes isolated from crops (such as wheat, barley, maize, and rice) also show a relationship with plant stress resistance (Wang et al., 2016; Wang et al., 2017). A reduced cuticle permeability and lower susceptibility to water deficit was observed in CER1‐overexpression in *Arabidopsis* plants, but CER1 overexpression increased the susceptibility to bacterial and fungal pathogens (Bourdenx et al., 2011). Overexpression of WXP1 in *Medicago truncatula* increased cuticular wax accumulation and enhanced drought tolerance (Zhang et al., 2005). Therefore, understanding both the wax biosynthesis pathway and the identification of wax genes involved in stress tolerance is expected to have great potential for crop improvement by gene manipulation.

Faba bean (*Vicia faba* L), an annual grain legume, is widely distributed all over the world. Previous studies have shown that light environments, such as shading and canopy light transmittance, alter photosynthetic characteristics, grain yield, and disease resistance of faba bean (Ma, Dong, Zhu, & Dong, 2019, Wang, Xia, & Hua, 2007). Some studies of cuticular wax in faba bean have focused on wax composition in flowers or other organs (Griffiths, Graeme, Shepherd, & Gavin, 1999; Zhao et al., 2019), yet few report the influence of light on the properties and functions of cuticular wax in faba bean. Therefore, in this study, the phenotypic plasticity of cuticular waxes to light wavelengths was analyzed, including white, red, yellow, blue, and purple lights provided by LEDs. Next, RNA‐seq and transcriptome processing were applied to analyze molecular responses to light wavelengths. We addressed the following questions: (1) How do the chemical profiles of faba bean cuticular wax respond to light wavelengths? (2) Is the expression of wax‐related genes regulated by light wavelengths? (3) Will the alterations of cuticular waxes induced by light wavelengths influence the leaf eco‐physiological functions?

**Core Ideas**

- Leaf cuticular waxes are sensitive to different light wavelengths.
- Cuticular waxes of faba bean were subjected to white, red, yellow, blue, and purple light to analyze phenotypic plasticity and function.
- Candidate genes involved in cuticular wax biosynthesis were identified by RNA‐seq and transcriptome processing.
- Wavelengths differentially altered wax coverage, eco‐physiological function, and gene expression levels.
- Light wavelengths may be applied in selecting candidate genes involved in wax biosynthesis in other crops.

## 2 MATERIALS AND METHODS

### 2.1 Plant growth conditions

The study was carried out in a greenhouse at Southwest University, Chongqing, China. Faba bean seeds (*Vicia faba* cv Qidou 2; supplied by Qin Yuan Chun Seeds Co., Ltd., Huaian, China) were surface sterilized with 3% H_2_O_2_, cleaned under tap water, then sowed in pots (18 cm height, 23 cm diameter) filled with a mixture of soil, peat, and vermiculite (2:1:1). The temperature of the glasshouse was 25/20 °C (day/night) and the relative humidity ranged from 65 to 75%. The positions of the pots were adjusted every three days to make sure that the plant tops received 50 μmol m^−2^ s^−1^ PPFD during a photoperiod of 12/12 h (light/dark).

### 2.2 Experimental design

**FIGURE 1:**
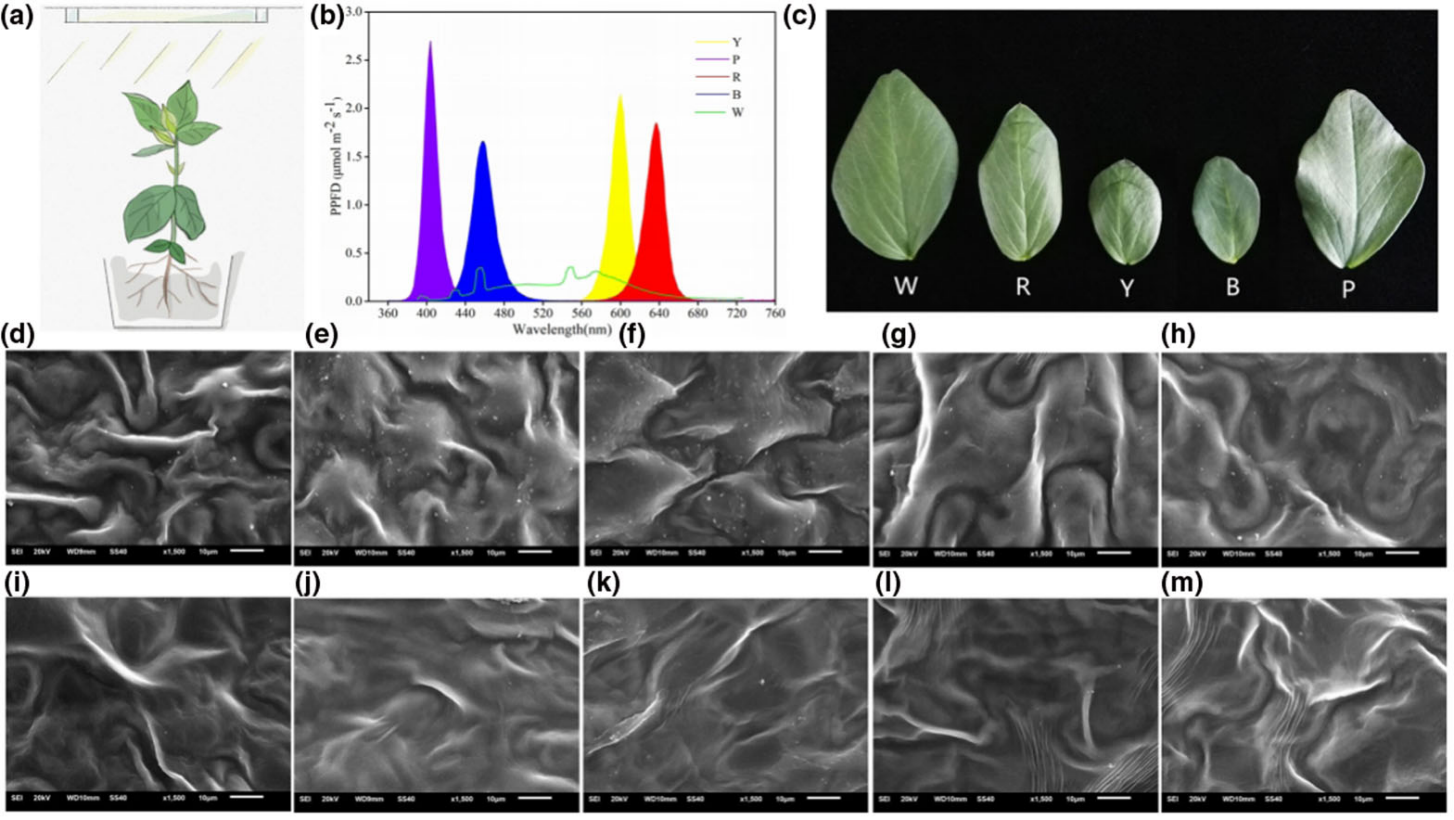
Phenotype of faba bean leaves grown under different light treatments. (a) Plants grown in greenhouse under LEDs light. (b) Spectral irradiance of the different light sources used in the experiments. (c) Leaf morphology under white (W), red (R), yellow (Y), blue (B) and purple (P) lights, respectively. (d‐h) Cuticular wax crystallization patterns of adaxial leaf surface under white (W), red (R), yellow (Y) blue (B), and purple (P) lights, respectively (Scale bars: 10 μm). (i‐m) Cuticular wax crystallization patterns of abaxial leaf surface under white (W), red (R), blue (B), yellow (Y) and purple (P) lights, respectively (Scale bars: 10 μm)

In total, there were four light wavelengths obtained from LEDs light, including red (637 nm), yellow (600 nm), blue (458 nm), and purple (403 nm); white light was the control. Light spectra were measured with Hipoint HR‐350 spectrometer and were shown in Figure 1b. Each light treatment was replicated four times. One week after seed germination, the number of plants per pot was thinned to six seedlings. Black light‐proof cloth was placed between different treatments to avoid light disturbance. One month later when faba bean were in four‐leaf stage, leaves were sampled for the following measurements.

### 2.3 Photosynthesis assays

Photosynthesis parameters were measured between 9:00 AM to 11:00 AM from the fourth fully expanded leaf by a portable photosynthesis measuring system (LCpro‐SD) with the photosynthetic photon flux at 1000 μmol m^−2^ s^−1^. The photosynthesis parameters included photosynthesis rate (Pn), transpiration rate (Tr), stomatal conductance (gs), and intercellular CO_2_ concentrations (Ci).

### 2.4 Wax extraction and GC‐MS analysis

The leaves (the fourth fully expanded leaf) were sampled, cleaned gently under ultrapure water, and photographed to measure their surface area using ImageJ software before wax extraction (Abràmoff, Magalhães, & Ram, 2004). The leaf samples were extracted twice for 30 s in 4 ml of chloroform containing 5 μg *n*‐Tetracosane as internal standard at room temperature. The two extracts from each sample were then combined and filtered through glass wool, the solvent was evaporated under a gentle stream of N_2_ gas while at 40 °C, and then derivated with 20 μl pyridine and 20 μl bis‐N,O‐trimethylsilyltrifluoroacetamide (BSTFA) for 45 min at 70 °C. The surplus reagents were evaporated under N_2_. The extract was dissolved in 200 μl of CHCl_3_ for gas chromatography (GC) analysis (Buschhaus & Jetter, 2012).

The GC analysis was conducted according to the method described by Ni et al. (2014c) with slight modification. The GC column was a 30 m by 0.25 mm by 0.2 μm DM‐5 capillary column and the carrier gas was nitrogen. The injector and flame ionization detector (FID) temperature were set at 300 °C and 320 °C, respectively. The oven temperature for the GC was programmed with an initial temperature of 80 °C and raised by 15 °C min^−1^ to 260 °C, and held for 10 min at 260 °C. The temperature was then increased at 5 °C min^−1^ to 320 °C, and held for 15 min at 320 °C. Compound identification was based on the mass spectra obtained from GCMS‐QP2010 Ultra Mass Spectrometric Detector (Shimadzu Corp., Kyoto, Japan) and published mass spectra (Zhao et al., 2019). Cuticular wax quantification (μg cm^−2^) was based on the FID peak area as compared with the internal standard peak area.

### 2.5 Scanning electron microscopy (SEM)

The fresh leaf samples (the fourth fully expanded leaf) of plants under different light wavelengths were collected, rinsed gently with ultrapure water, and then air‐dried. Segments of 4 mm^2^ were sampled from the center of leaves, affixed on an aluminum stub, coated with foil, and then viewed by SEM (Hitachi S3500 and JSM‐6510LV) (Ni, Sun, Huang, Huang, & Guo, 2015b).

### 2.6 Water loss and stomatal index

The plants grown under different light wavelengths were dark acclimated for 12 h to ensure stomatal closure, and then the leaf samples were immersed into distilled water for 1 h and weighed. Next, the leaf samples were put into a dark chamber for continuous dehydration, weighed at 15‐min intervals for 150 min, dried at 70 °C for 24 h, and then weighed (Burkhardt & Pariyar, 2014). The water loss percentage was calculated as follows:

Water loss (%) = (saturated weight − fresh weight)/(saturated weight − dry weight) × 100

A linear model was applied between water loss percentage and time according to Pearson's correlation analysis using OriginPro 8.0.

Leaf abaxial epidermis of faba bean was torn off using tweezers to observe the number of pavement cells and stomata at 10 × 40 time magnification by a micro‐morphology. The number of pavement cells and stomata were counted from six views of each slide for one replicate (Visser et al., 1997). Stomatal index, an important indicator for leaf surface gas exchange that normalizes the effects of epidermal cells expansion, was calculated as follows:

Stomatal index = Stomata number/(cell number + stomata number) × 100

### 2.7 RNA‐seq and transcriptome data processing

The leaf samples from three lights (white, yellow, and purple) were sent to Biomarker (BMK, Beijing, China) for RNA extraction and sequencing, with three biological replicates. RNA concentration was measured using Nano Drop 2000 (Thermo), and RNA integrity was assessed using the RNA Nano 6000 Assay Kit of the Agilent Bioanalyzer 2100 (Agilent Technologies, CA, USA). The nine RNA‐Seq libraries and the raw reads were generated using the Illumina Hiseq2500 pair‐end sequencing.

Clean data were obtained by removing reads containing adapters, reads containing unknown bases (N), and low quality reads from raw data. *De novo* assembly was accomplished using the Trinity method in which min_kmer_cov was set to 2 by default and all other parameters were set to default (Grabherr et al., 2011). The reads with a certain overlap length were first combined to form longer fragments, which were called contigs. These reads were then mapped back to contigs, and the contigs and paired‐end reads from the same transcript were detected and the distance determined. Finally, these contigs were assembled to form unigenes that could not be extended on either end.

All assembled unigenes function was annotated based on the following databases: NR (NCBI non‐redundant protein sequences), Pfam (Protein family), KOG/COG/eggNOG (Cluster of Orthologous Groups of proteins), Swiss‐Prot (A manually annotated and reviewed protein sequence database), KEGG (Kyoto Encyclopedia of Genes and Genomes), and Go (Gene Ontology).

### 2.8 Analysis of differentially expressed genes (DEGs)

The expression levels of unigenes were estimated by RNA‐Seq by Expectation Maximization (RSEM) (Li & Dewey, 2011) for each sample. Differential expression analysis of two conditions was performed using the DESeq R package (1.10.1). DESeq provide statistical routines for determining differential expression in digital gene expression data using a model based on the negative binomial distribution. The resulting P values were adjusted using the Benjamini and Hochberg's approach for controlling the false discovery rate (FDR). Unigenes with an adjusted P‐value < 0.05 found by DESeq were assigned as differentially expressed. Multiple alignment analyses and expression heat‐map of some DEGs involved in the light response pathways were selected and performed using complete linkage method and hierarchical with genes by the Cluster 3.0, and TreeView‐1.1.6r4 was used for image display. According to the FPKM values of these differentially expressed genes, the correlation coefficients were calculated by Excel 2010 and gene co‐expression network was finally performed by Cytoscape_V3.7.0.

### 2.9 Wax‐related unigenes selection and quantitative real‐time reverse transcription‐PCR (qRT‐PCR) expression analysis

According to the DEGs functional annotation, we preliminarily selected 34 likely wax‐related unigenes, which were involved in the VLCFAs elongation, alcohol‐forming, and alkane‐forming pathways. Next, we performed the primary function prediction of these unigenes, including ORF and conserved domain of encoded amino acid sequences. Finally, ten unigenes, which have the typical conserved domain of protein, belonging to the known wax gene families were chosen. The amino acid sequences of these 10 unigenes were compared with the reported homologous genes from 27 dicotyledon plants in NCBI database, especially leguminous plants, followed by phylogenetic analysis. In addition, qRT‐PCR was used to validate the responses of wax genes expression to different light wavelengths.

Total RNA was extracted from the faba bean leaves grown under different light wavelengths using EZ‐10 DNAaway RNA Mini‐preps kit (BBI life science, China), and cDNA was prepared from it using Primescript RT reagent kit with gDNA eraser (Takara, China). The primers used in this assay were listed in the supplemental Table S3. qRT‐PCR were performed as follows: 95 °C for 30 s, 39 cycles of 95 °C for 5 s, 55–65 °C for 30 s, and 65 °C for 5 s (Tm was listed in Supplemental Table S3). The 18s gene was used as an internal control, and the transcript level in faba bean leaves grown under white light was set as 1.0. The relative expression levels of these targeted unigenes were calculated with the 2^−△△Ct^ method. Three biological and four technical replicates were performed per sample.

### 2.10 Statistical analysis

All statistical analyses were performed using SPSS version 19.0 (SPSS, Inc., Chicago, IL, USA). The data were the mean of four replicates. One‐way ANOVA was applied to estimate the effects of light wavelengths on the property of cuticular wax, and the significant differences between light wavelengths were tested according to the least significance difference (LSD) test. False discovery rate (FDR) correction was also applied, with *P* < .05 as the statistical significance threshold.

## 3 RESULTS

### 3.1 Morphological and chemical responses of cuticular waxes to different light wavelengths

**FIGURE 2:**
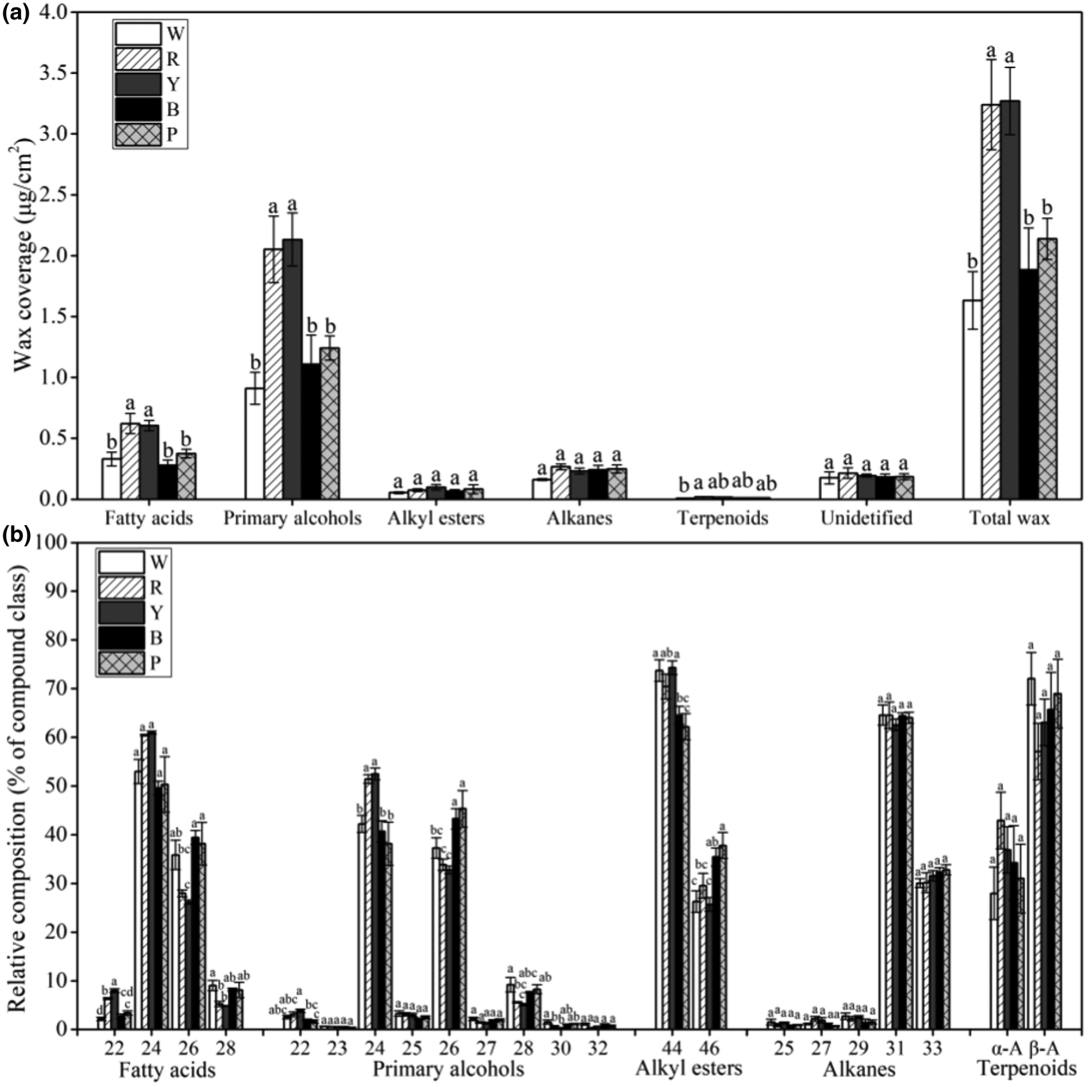
Cuticular wax compositions and amounts in leaves of faba bean grown under different light treatments. (a) The composition and content of wax in faba bean leaves. (b) The chain lengths distribution of wax compounds. W, R, Y, B and P represent white, red, yellow, blue and purple light, respectively. The error bar represents standard error. Different lowercase letters above data bars within each compound class represent significance at *P* < 0.05 according to the least significant difference test

One month after growing under different light wavelengths, faba bean showed obvious morphological changes of leaf and cuticular waxes (Figure 1c‐1m ). The size and shape of leaves varied greatly between five light wavelengths, with leaf area ranging from 13.48 cm^2^ under yellow light to 49.41 cm^2^ under white light. The SEM analysis also indicated that wax film covered leaf surface with platelet‐like wax crystalloids sporadically distributed. Overall, more platelet‐like wax crystalloids could be observed on adaxial leaf surface under yellow light when compared with other light wavelengths. In total, five classes of wax compounds were identified in faba bean leaves, including fatty acids, primary alcohols, alkyl esters, alkanes, and terpenoids (Figure 2a). Overall, primary alcohols were the predominant wax class (61.21%), followed by fatty acids (18.13%) and alkanes (9.39%), with about 7.74% of unidentified compounds. Among the different light wavelengths, the total wax coverage under red (3.24 μg cm^−2^) and yellow (3.27 μg cm^−2^) lights were significantly higher than those under other lights, with insignificant difference between white, blue and purple lights. The responses of the contents of fatty acids and primary alcohols to different light wavelengths were similar to total wax coverage. The contents of alkanes under white light were lower than those under monochromatic lights. No significant difference in the contents of alkyl esters and unidentified components was observed under different light wavelengths.

The five identified wax classes were consisted of series of homologs with different chain lengths (Figure 2b). The chain length of fatty acids ranged from C_22_ to C_28_, with C_24_ the predominance (45.02%). The relative abundance of C_22_ and C_24_ acids under yellow light was higher than those under other lights, whereas the relative abundance of C_26_ and C_28_ under yellow light was lower than other lights. Primary alcohols were consisted of both even and odd carbon numbers with chain length ranging from C_22_ to C_32_, which exhibited significant odd/even predominance with the relative abundance of even carbon numbers reached up to 94.93%. Different light wavelengths imposed a direct influence on the predominant primary alcohol compound, with C_24_ under white, red and yellow lights and C_26_ under blue and purple lights. Two alkyl esters, C_44_ and C_46_, were identified with C_44_ the predominant compound. The alkanes were consisted of odd carbon number compounds with chain length ranging from C_25_ to C_33_ and with C_31_ (64.03%) the predominant one. The terpenoids identified in faba bean leaves were β‐amyrin (65.37%) and α‐amyrin (34.63%). No significant difference in the relative abundance of alkanes and terpenoids could be observed under different light wavelengths. The average chain length (ACL) and carbon preference index (CPI) of fatty acids, primary alcohols and alkanes also showed no significant responses to different light wavelengths. (Supplemental Table S1).

### 3.2 Functional changes of cuticular wax in faba bean leaves

**FIGURE 3:**
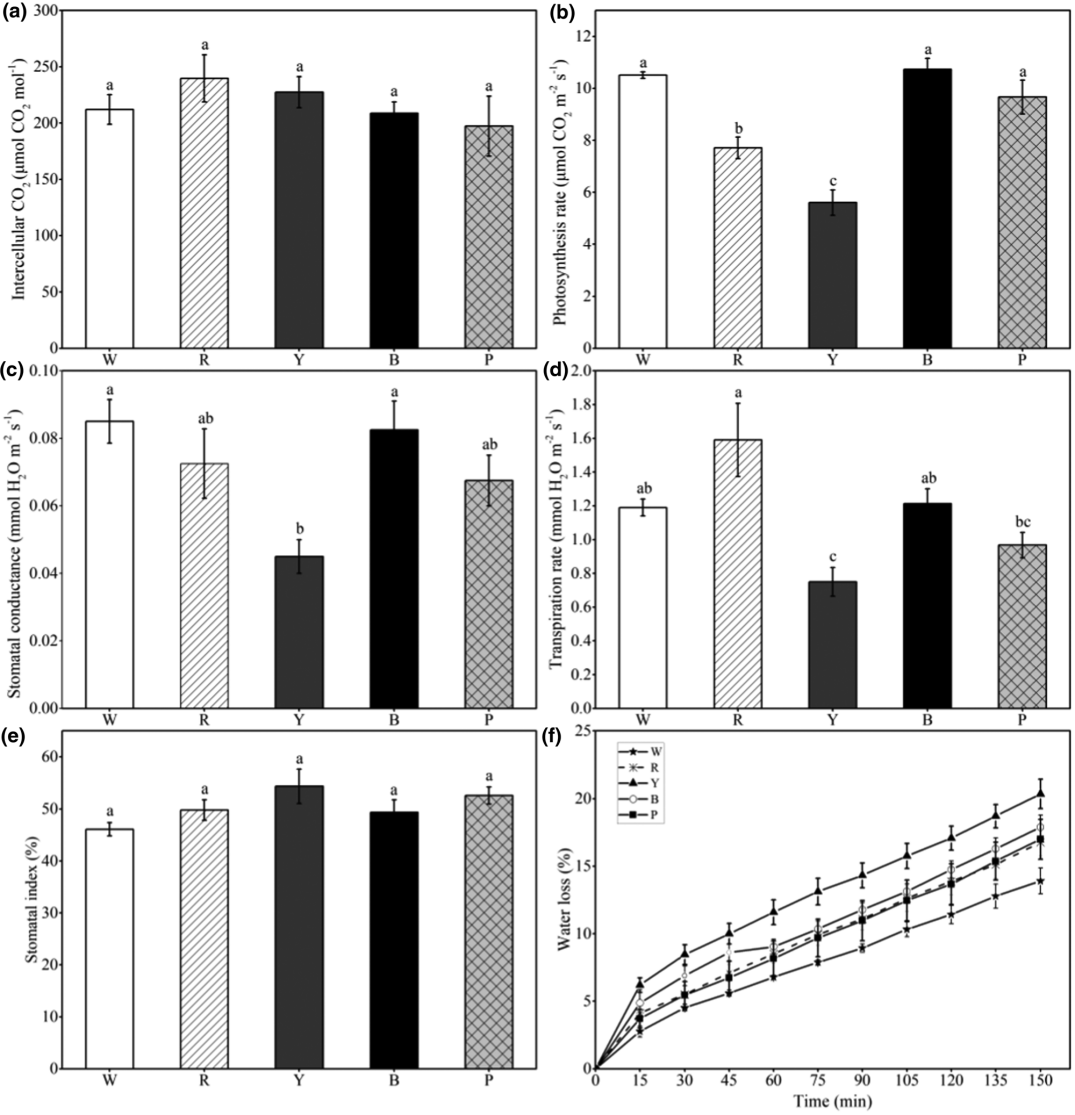
Functional changes of cuticular wax in faba bean leaves grown under different light treatments. (a‐d) Photosynthesis parameters of faba bean leaves under different light treatments. (e) Stomatal index of faba bean abaxial leaf under different light treatments. (f) Water loss of faba bean leaves under different light treatments. W, R, Y, B and P represent white, red, yellow, blue and purple lights, respectively. The error bar represents standard error. Different lowercase letters above data bars represent significance at *P* < .05 according to the least significant difference test

Photosynthesis parameters, leaf water loss and stomatal index were further measured. Compared to those under white light, the P_n_, Tr and gs under yellow light significantly decreased by 46.72, 36.97, and 47.06%, respectively (Figure 3). In addition, red light significantly decreased the Pn. No significant difference in stomatal index of abaxial leaf epidermis was observed under different light wavelengths. The leaf water loss was positively correlated with time (Supplemental Figure S1). Leaf water loss rate under white light was the lowest, which was significantly lower than those under yellow and purple lights.

### 3.3 Transcriptome sequencing of faba bean leaves under three light wavelengths

To test whether the genes involved in wax biosynthesis in faba bean leaves also differentially responded to light wavelengths, 9 leaf samples from white, yellow, and purple lights were subjected to Illumina Hiseq paired‐end reads. The correlation analysis among these 9 samples was shown in Supplemental Figure S2. In total, approximately 59.69 Gb of clean data was obtained, and 100,194 unigenes were assembled using Trinity, which were mainly distributed in N50 length of 1,281 bp and mean length of 732.99 bp. Among them, 8,244 (8.23%) unigenes were more than 2,000 bp in length (Table 1). All reads of sequencing were submitted to the NCBIs Short Read Archive (accession number SRR8874301‐SRR8874309).

**TABLE 1:** Summary of transcriptome assembly for faba bean

	Total length (percentage)	
Length range	Transcripts	demo. Unigene
200–300	36382 (18.28%)	33332 (33.27%)
300–500	37920 (19.06%)	29244 (29.19%)
500–1000	41563 (20.89%)	18608 (18.57%)
1000–2000	45735 (22.98%)	10766 (10.75%)
2000+	37385 (18.79%)	8244 (8.23%)
Total number	198,985	100,194
Total length	235,496,841	73,441,015
N50 length	1968	1281
Mean length	1183.490419	732.988153

For the validation and annotation of the assembled unigenes, all the unigenes were searched against the COG, Go, KEGG, KOG, Pfam, Swiss‐Prot, eggNOG, and NR databases using BLAST and HMMER programs. In total, 54,500 (54.39% of all unigenes), 51,683 (51.59%), 37,707 (37.63%), 35,769 (35.70%), 32,914 (32.85%), 32,852 (32.79%), 21,209 (21.17%) and 16,963 (16.93%) unigenes were found in the eggNOG, NR, Pfam, Go, KOG, Swiss‐Prot, KEGG and COG databases, respectively. Overall, 60,614 unigenes were annotated in the aforementioned databases (Supplemental Table S2).

### 3.4 Analysis of differentially expressed genes (DEGs)

The transcript abundance of unigenes in faba bean leaves under different light wavelengths was estimated by fragments per kilobase of transcript per million mapped reads (FPKM). Using the DESeq 2 and FDR < 0.01 and FC ≥ 4 as criterion, 1,187 DEGs were identified, of which 583 up‐regulated and 604 down‐regulated genes were observed in purple light. In yellow light, 2,159 DEGs were observed, including 978 up‐regulated and 1,181 down‐regulated genes. In addition, 3,252 unigenes showed significant difference in expression between yellow and purple lights (Table 2).

**TABLE 2:** Summary of differentially expressed genes (DEGs)

	ALL DEG	up‐regulated	down‐regulated
W vs P	1187	583	604
W vs Y	2159	978	1181
Y vs P	3252	1935	1317

### 3.5 Functional annotation of DEGs

**FIGURE 4:**
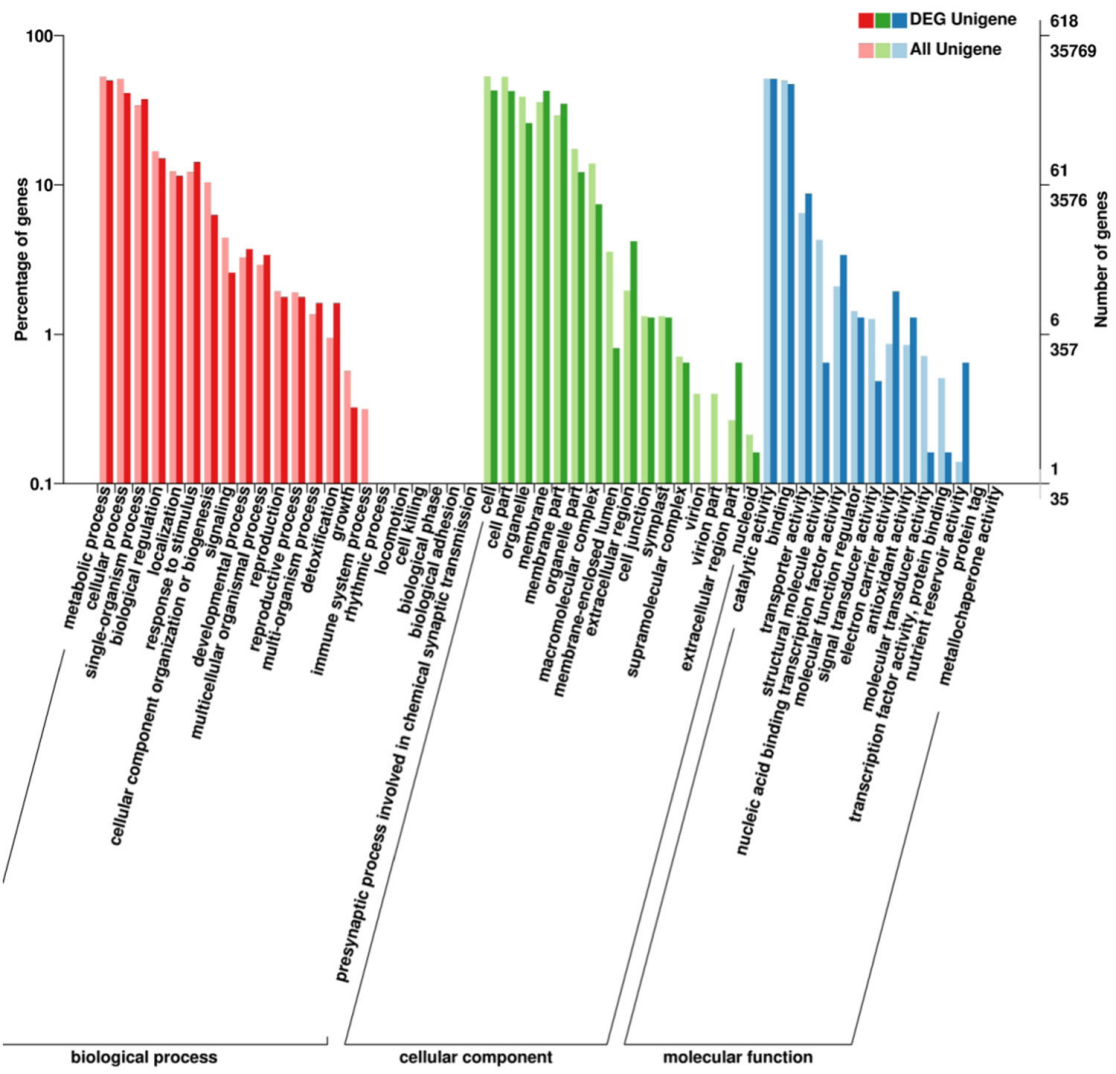
Histogram presentation of the Go classification of the DEGs between white and yellow lights. The results were summarized in three main categories, biological process, cellular component, and molecular function. The left y‐axis indicates the percentage of genes in a category, and the right y‐axis indicates the number of genes in a category

There were 257, 618, and 1,178 DEGs being assigned with one or more Go terms at white vs purple, white vs yellow, and yellow vs purple lights, respectively (Figure 4; Supplemental Figure S3). The most represented Go terms were biological process, cellular component and molecular function. In biological process, metabolic process was the most abundant, and some DEGs were observed in “biological regulation”, “signaling”, or “response to stimulus” and “growth”. DEGs were mainly observed in the “membrane”, “membrane part” and “cell part” in the cellular component category, and “catalytic activity” and “binding” in the molecular function category, respectively. Different enrichment trends of all unigenes and DEGs were also observed in “extracellular region” and “extracellular region part” in the cellular component category, “nutrient reservoir activity” and “electron carrier activity” in the molecular function category.

**FIGURE 5:**
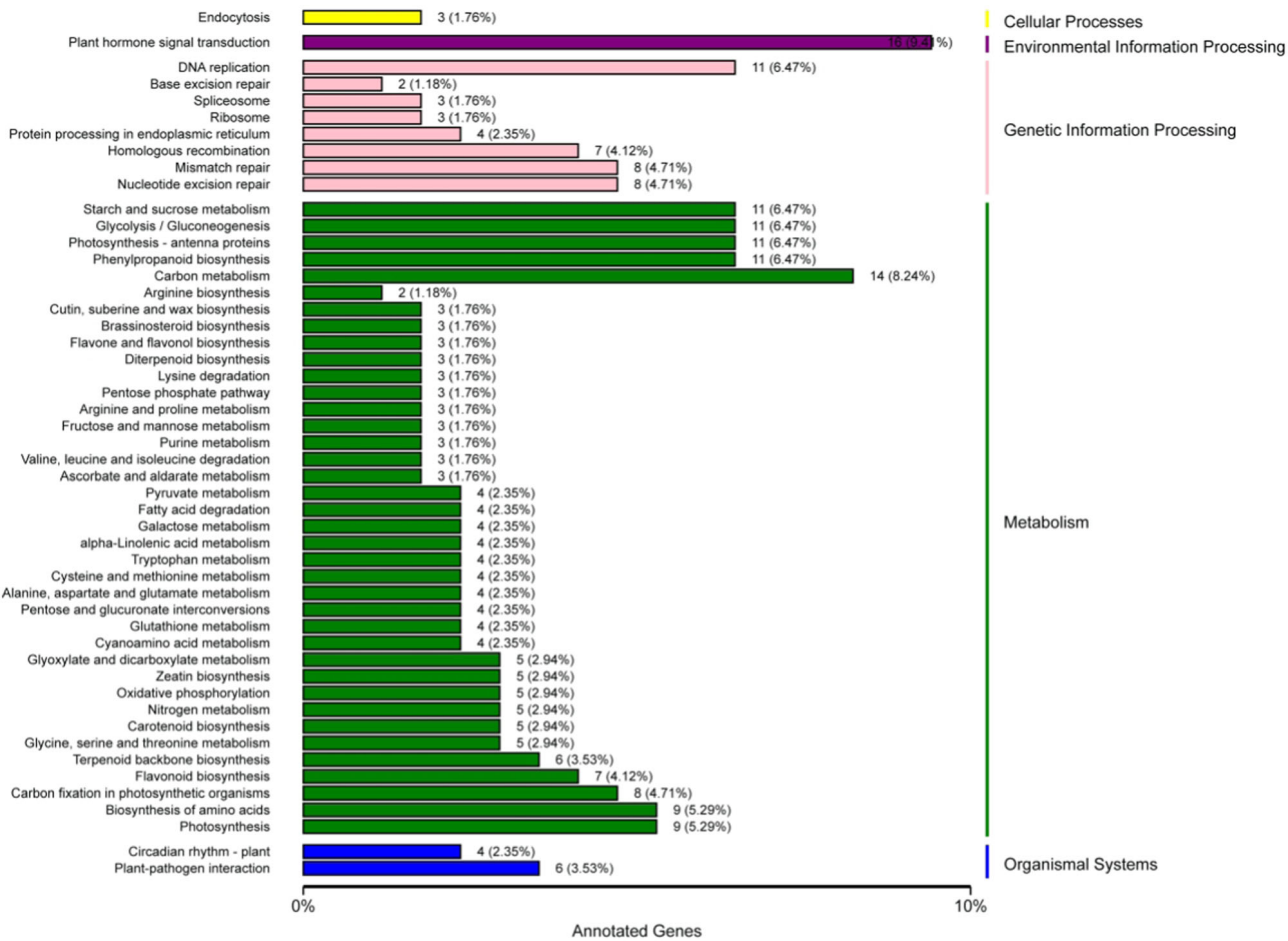
KEGG pathway enriched for DEGs in faba bean leaves between white and yellow lights. The annotated genes are the ratio of the number of DEGs annotated in a given pathway term to the number of all genes annotated in the pathway term. The top 50 enriched pathway items in the KEGG database were listed. Blue bar: classification based on organismal systems categories. Green bar: classification based on metabolism categories. Pink bar: classification based on the genetic information processing. Purple bar: classification based on the environmental information processing categories. Yellow bar: classification based on cellular processes categories

To further understand the functions of DEGs, DEGs were also subjected to a search against the KEGG database for further functional prediction and classification (Figure 5; Supplemental Figure S4). As a result, the “plant hormone signal transduction” and “carbon metabolism” were the most abundant pathways. In addition, some DEGs were involved in the biosynthetic pathways for “cutin, suberin, wax, flavone, flavonol, and terpenoid” and “fatty acid degradation”, these might attributing to the varied deposition of cuticular wax under different light wavelengths.

### 3.6 Expression analysis of DEGs under different light wavelengths

**FIGURE 6:**
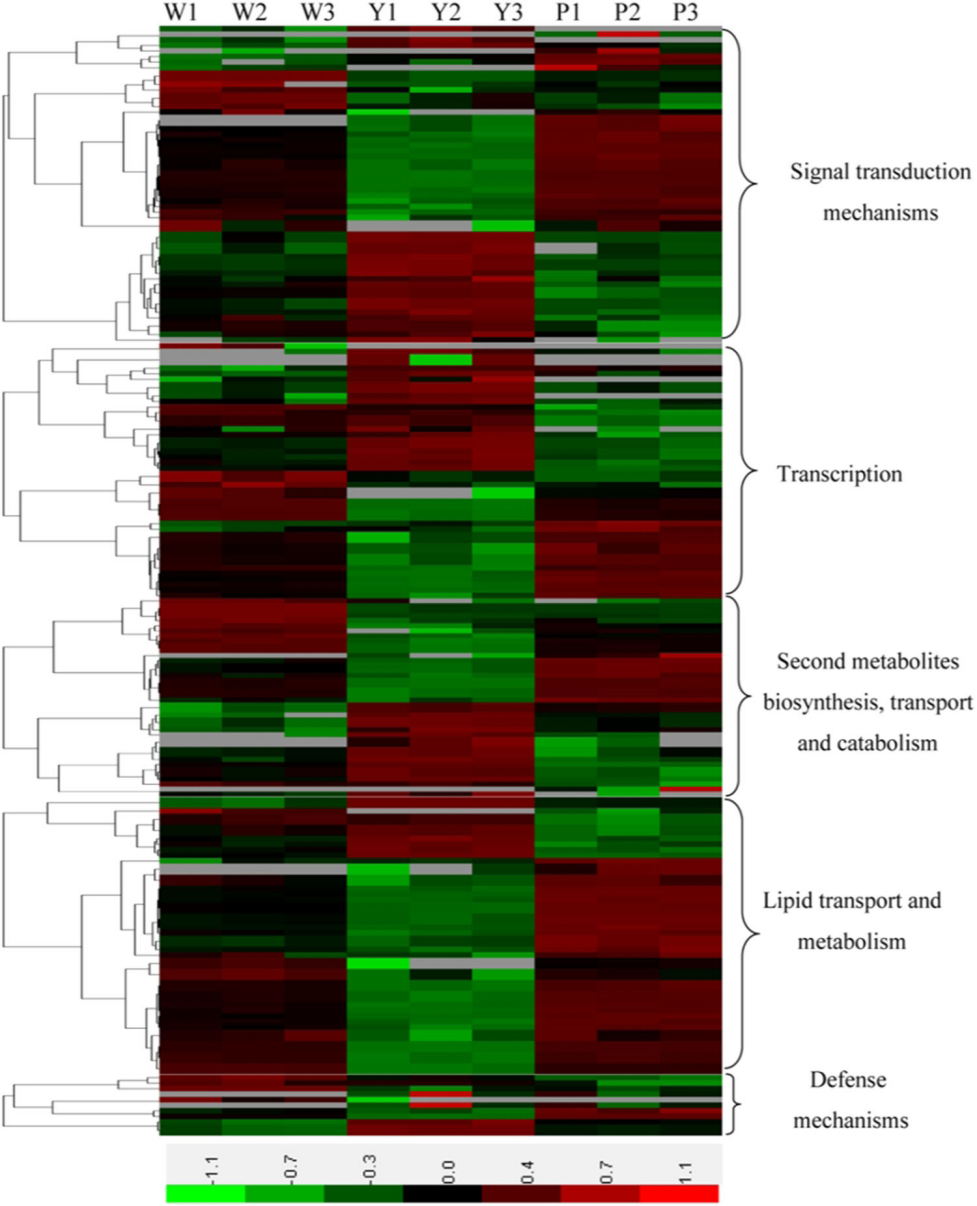
Differential expression of unigenes involved in some pathways likely respond to the changing light treatments. The clustering was computed using complete‐linkage method. The columns in heatmap represent different samples, the row represent different unigenes involved in specific pathways. W1, W2, and W3 represent three biological replicates of white light; Y1, Y2 and Y3 represent three biological replicates of yellow light; P1, P2 and P3 represent three biological replicates of purple light. Color scale represents log2 fold change values. The green refer to a decrease in expression, red refer to an increase in expression

**FIGURE 7:**
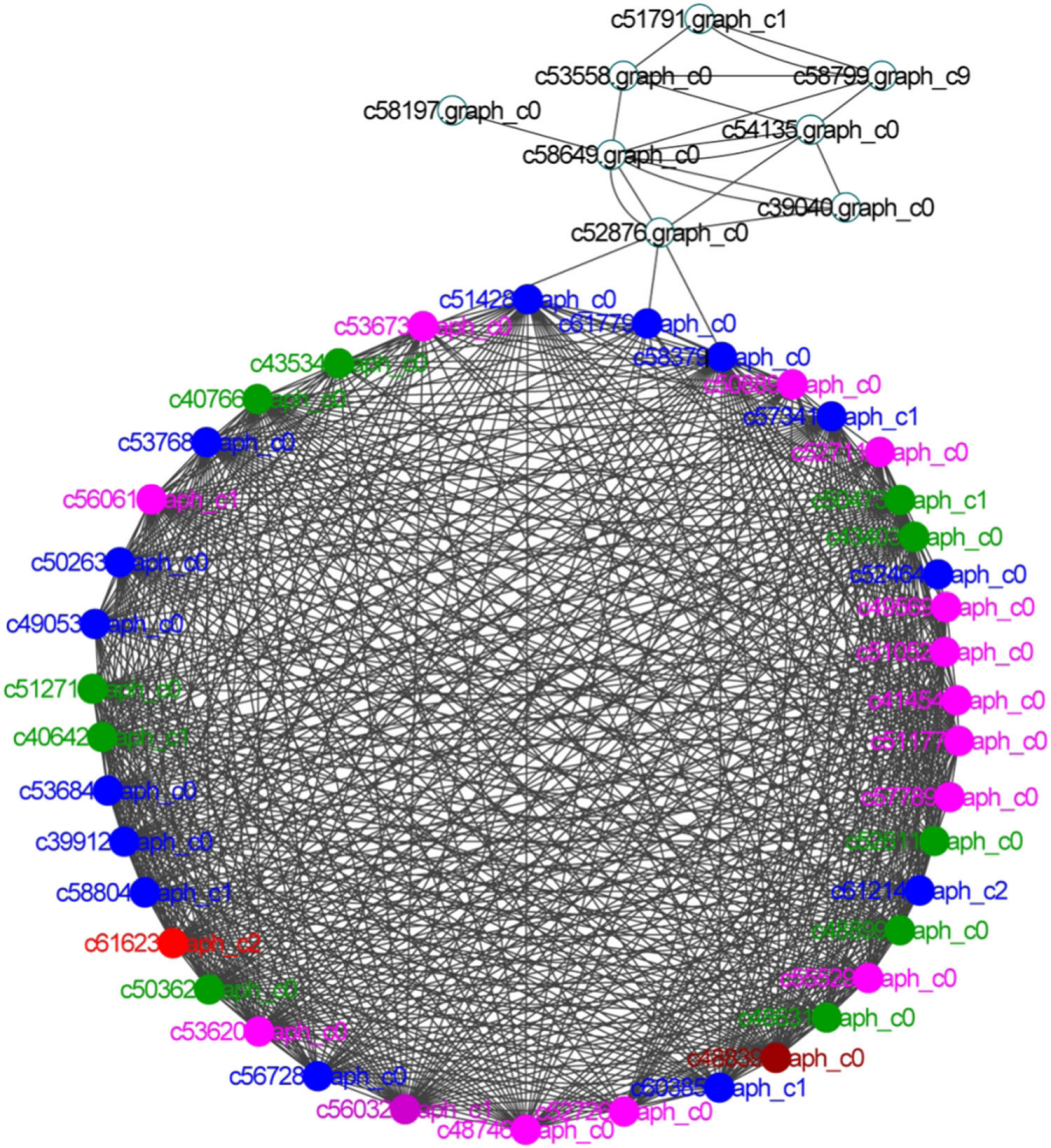
Co‐expression network of DEGs involved in different pathways. Circles represent genes, and edges between two circles represent interactions between genes. Blue circles represent genes involved in signal transduction pathway; green circles represent genes involved in second metabolites biosynthesis, transport and metabolism pathway; purple circles represent genes involved in lipid transport and metabolism pathway, red circles represent genes involved in defense mechanisms

To understand the influence of changing light wavelengths on the expression level of genes, we selected some DEGs involved in the five likely pathways and carried out the expression profile analyses, such as signal transduction mechanisms (45 annotated DEGs), transcription (35 annotated DEGs), second metabolites biosynthesis, transport and catabolism (30 annotated DEGs), lipid transport and metabolism (34 annotated DEGs), and defense mechanisms (9 annotated DEGs). The expression patterns of some genes responded to light wavelengths showed high consistency in different pathways (Figure 6). Furthermore, we performed correlation analysis according to the FPKM values of DEGs expression involved in these five pathways and selected the unigenes with absolute correlation coefficient greater than 0.9 (49 DEGs) for co‐expression analyses. The results showed that 40 DEGs presented very similar expression profile and interacted each other (Figure 7), suggesting that these genes collectively responded to the change of light wavelengths.

### 3.7 Candidate DEGs involved in wax biosynthesis in faba bean leaves

**FIGURE 8:**
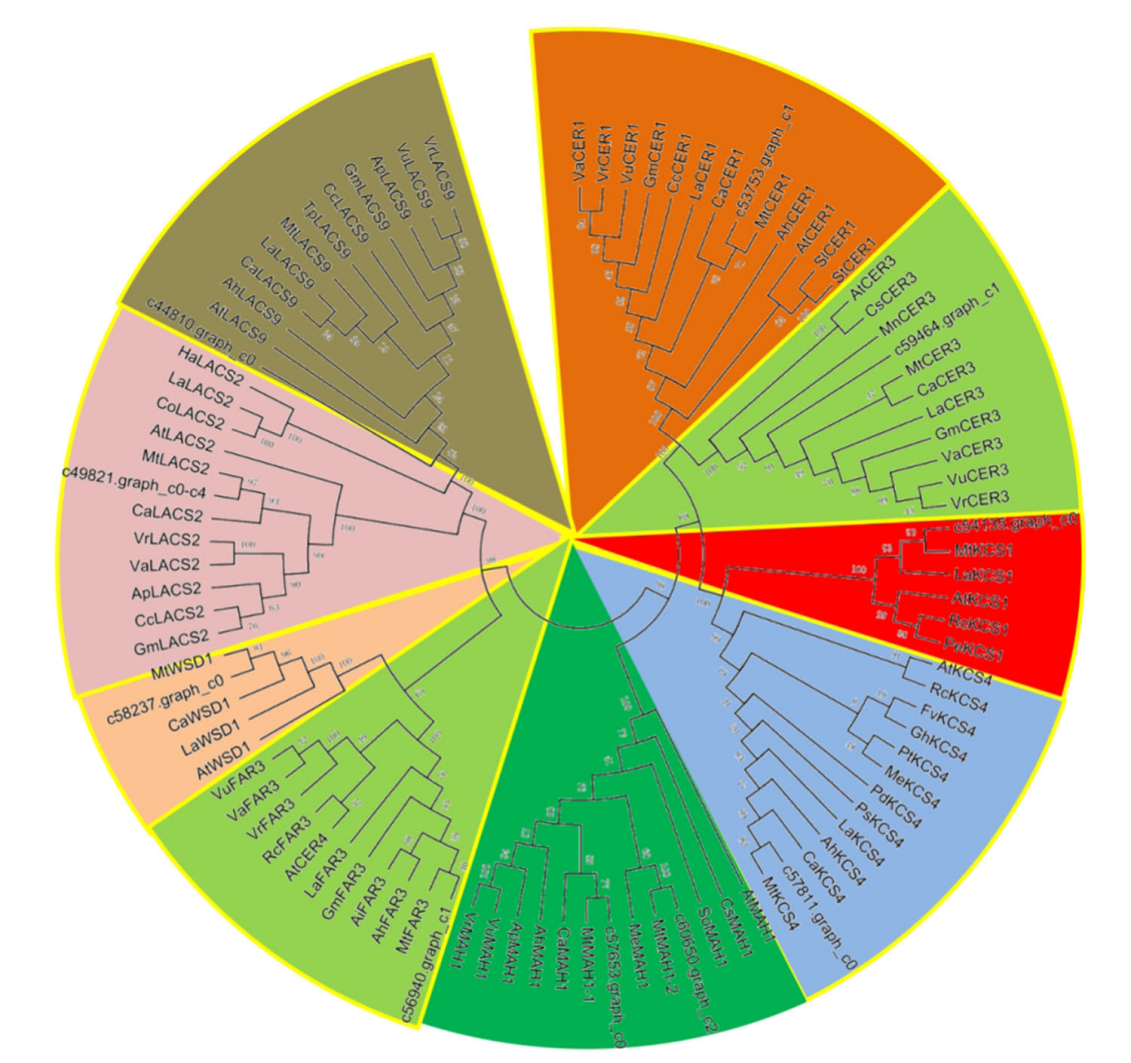
The cladogram and classification of 10 wax‐related gene families. The neighbor‐joining (NJ) tree represented relationships among 100 wax‐related genes from 27 species. Numbers by the branches represented bootstrap values from 1000 neighbor‐joining (NJ) analysis. Proteins were classified into 10 families, each colored pie slice encompassed the same waxy genes annotated in this family obtained from other published plant species

To investigate the functional genes involved in wax biosynthesis in faba bean leaves, we preliminary screened wax‐related unigenes according to the annotation of known databases and predicted the ORF and amino acid sequences of these unigenes. In total, 10 unigenes annotated in the wax biosynthesis were obtained. A Neighbour‐joining (NJ) tree was constructed using MEGA 5.10, based on the alignment between these unigenes and the functional genes known in biosynthesis of cuticular wax from 27 different species (including leguminous crop and forage). Phylogenetic and functional analyses revealed that these selected unigenes in faba bean were classified into different wax biosynthesis pathways: VLCFAs elongation, primary alcohol, wax ester and alkane formation, and the secondary alcohol and ketone formation (Figure 8). DNA similarity between *Medicago truncatula* (leguminous model plant) and faba bean reached over 60%, and their amino acid similarity reached over 69%. The unigenes obtained from faba bean were the putative homologous of LACS2, LACS9, KCS1, KCS4, FAR3, WSD1, CER1, CER3, MAH1‐1 and MAH1‐2 in *M. truncatula* (Table 3).

**TABLE 3:** Summary of unigenes involved in the wax biosynthesis in faba bean leaves under different wavelengths of light

Clone No	Unigene ID	Gene name	Accession No	Species which have the highest identity from NCBI	Sequence ID	DNA similarity	Protein similarity
1	c49821.graph_c0,c1,c3,c4	LACS2	MK775520	*Medicago truncatula*	XM_013611275.2	83%	90%
2	c44810.graph_c0	LACS9	MK775021	*Medicago truncatula*	XM_003589534.3	83%	87%
3	c54135.graph_c0	KCS1	MK775518	*Medicago truncatula*	XM_003626553.3	79%	93%
4	c57811.graph_c0	KCS4	MK775519	*Medicago truncatula*	XM_003606879.3	78%	94%
5	c56940.graph_c1	FAR3	MK775517	*Medicago truncatula*	XM_003596735.3	61%	83%
6	c58237.graph_c0	WSD1	MK775524	*Medicago truncatula*	XM_003588474.3	80%	84%
7	c53753.graph_c1	CER1	MK775515	*Medicago truncatula*	XM_003626786.3	86%	92%
8	c59464.graph_c1	CER3	MK775516	*Medicago truncatula*	XM_013603097.2	81%	87%
9	c57653.graph_c0	MAH1‐1	MK775522	*Medicago truncatula*	XM_013611326.1	80%	69%
10	c60650.graph_c2	MAH1‐2	MK775523	*Medicago truncatula*	XM_013595291.2	82%	78%

### 3.8 qRT‐PCR validation of candidate DEGs involved in wax biosynthesis

**FIGURE 9:**
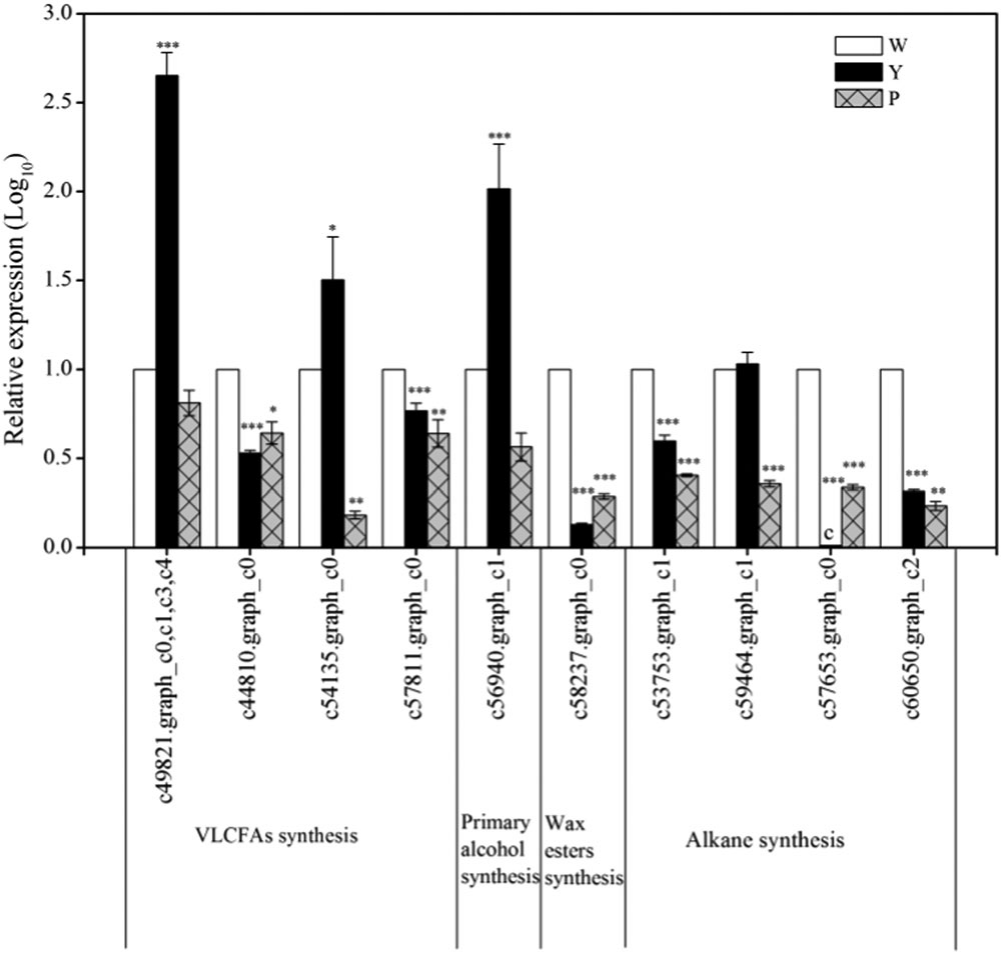
Validation of 10 unigenes under different light treatments involved in wax biosynthesis by qRT‐PCR. W, Y and P represent white, yellow and purple lights, respectively. Asterisks indicate levels of significance of differential expression (* *P* < .05,** *P* < .01, *** *P* < .001)

To validate the responsible genes involved in the wax biosynthesis, the expression levels of 10 candidate DEGs from faba bean were evaluated by qRT‐PCR. Light imposed a significant difference on the wax‐related gene expression between different wavelengths (Figure 9). LACS2, KCS1 and FAR3 were dramatically up‐regulated by yellow light, whereas some genes involved in the decarbonylation pathway (CER1, MAH1) were significantly down‐regulated by both yellow and purple lights.

## 4 DISCUSSION

Plant leaf cuticular wax alters its deposition or compositions to adapt to changing environments (Guo, Gao, He, & Guo, 2016). In this study, we also observed that light wavelengths influenced the wax deposition and composition on faba bean leaves. For example, the total wax coverage under red and yellow lights was significantly higher than those under other lights. More platelet‐like wax crystalloids were observed on adaxial leaf surface under yellow light. In hence, we speculated that certain biological process might be required to support the morphological alterations of cuticular wax in faba bean leaves under different light wavelengths. To support this speculation, a comparative de‐novo transcriptome analysis of faba bean leaves under different light wavelengths was conducted.

In total, 100,194 unigenes (N50 = 1281 bp) was assembled by Trinity, of which 19,010 (18.97%) unigenes were greater than 1 kb. The number of unigenes obtained in this study was in consistent with the previous faba bean transcriptome assemblies where the unigenes ranged from 42,915 to 164,647 (Cooper et al., 2017; Kaur et al., 2012; Khan et al., 2019; Ray, Bock, & Georges, 2015). The function of DEGs by Go and KEGG annotations mainly related to “signaling” and “response to stimulus” in the biological process category. This is in consistent with the functions of light which not only provides energy for photosynthesis, but also acts as a signal influencing the growth and development of plant through photoreceptors (Budde & Randall., 1990). Some DEGs were mainly annotated with “cutin, suberine and wax biosynthesis” and “terpenoids backbone biosynthesis” in KEGG database, suggesting that different expression of some genes involved in this pathway might be required to support cuticular wax alterations. This was also consistent with the altered terpenoids contents among different light wavelengths in this study.

Different wax components are synthesized by elongation of saturated fatty acyl chains and their modification (Kunst et al., 2006). As a necessary step in wax biosynthesis, saturated C_16_ and C_18_ fatty acid hydrolyzed from the acyl carrier protein (ACP) must be esterified to CoA by LACS (Jetter & Kunst, 2008; Lü et al., 2009). In this study, we obtained two LACS gene family members, LACS2 and LACS9, which varied their relative expression level between different light wavelengths. Characterization of the *lacs2* and *lacs9* mutant revealed that the mutation had no effect on the cuticular wax load or composition, and the export of acyl groups from the plastid (Schnurr, Shockey, & Browse, 2004; Kunst et al., 2006). The identification of these two LACS isozymes involved in the CoA esterification of fatty acid required for wax production remains to be further studied.

Elongation of C_16_ and C_18_ fatty acids to VLCFAs (C_20_ to C_34_) in ER is carried out by fatty acid elongases (FAE). In *Arabidopsis*, all FAE complexes share the same KCR, HCD and ECR enzymes, but their KCS components may differ and are thought to determine substrate and product chain length specificities of the overall complex (Zheng et al., 2005). In this study, we identified two KCS genes, KCS1 and KCS4. Gene expression analysis revealed that yellow light significantly up‐regulated the KCS1 expression level and down‐regulated the expression level of KCS4. In *A. thaliana*, complete loss of KCS1 expression resulted in decreases of up to 80% in the levels of C_26_ to C_30_ wax alcohols and aldehydes (Todd, Post‐Beittenmiller, & Jaworski, 2010), however, the loss of GLOSSY8 (which encodes a β‐ketoacyl reductase involved in wax biosynthesis) expression in maize resulted in 70% reduction in total surface waxes (mainly aldehydes and alcohols) (Bianchi, Avato, & Salamini, 1979). Phylogenetic analysis demonstrated that 21 KCS proteins formed eight distinct subclasses, and heterologous expression in *Saccharomyces cerevisiae* showed that only 8 among the 21 KCS proteins were found to be enzymatically active, including KCS9 and KCS17 of subclass α, while no activity of KCS4 (another component of subclass α) has been detected (Blacklock & Jaworski, 2006; Joubès et al., 2008). Our results indicated that increased expression of KCS1 under yellow light was accompanied by a dramatic increase in the contents of C_22_ to C_26_ wax alcohols and fatty acids. In hence, we speculated that KCS1 might be mainly involved in the biosynthesis of VLCFAs with chain lengths ranging from C_22_ to C_26_.

VLCFAs products are shared by more than one wax biosynthesis pathway. Primary alcohols were assumed to be produced by the reduction of fatty acyl‐CoA, which is catalyzed by fatty acyl‐CoA reductase (FAR). Studies of *cer4* wax‐deficient mutants of *Arabidopsis* demonstrated that FAR3 (CER4) was specifically involved in the production of very‐long‐chain primary alcohols with chain length ranging from C_24_ to C_28_, and heterologous expression of CER4 cDNA in yeast also resulted in the accumulation of C_24:0_ and C_26:0_ primary alcohols (Rowland et al., 2006). In this study, yellow light significantly increased the coverage of primary alcohols by 134.37%, especially the content of C_22_ to C_26_ alcohols (Supplemental Figure S5). Analysis of gene expression also revealed that yellow light significantly up‐regulated the expression level of FAR3. Wax ester, another product of acyl reduction pathway, which are esterified with fatty acids by WSD1 (Li et al., 2008). In this study, no significant difference was observed on the amount of wax esters in faba bean leaves under different light wavelengths. However, both yellow and purple lights down‐regulated the expression level of WSD1. Such inconsistency might be attributed to various biological functions of WSD1, which is also involved in the synthesis of triacylglycerol (Li et al., 2008).

The VLCFAs can also be modified into alkanes, secondary alcohols and ketones by the decarbonylation pathway. A new model for alkane production in which CER1 interacts with both CER3 and CYTB5 to catalyze the redox‐dependent synthesis of very long chain (VLC) alkanes from VLC acyl‐CoAs have been elucidated (Bernard et al., 2012). In this study, we also obtained CER1 and CER3 candidate unigenes. Light wavelengths differed their influences on the expression levels, with yellow light down‐regulated CER1 expression and up‐regulated CER3 expression, while purple light down‐regulated the expression level of both CER1 and CER3. However, both yellow and purple lights had no significant effects on the content and relative abundance of alkanes, suggesting the necessity of multiprotein enzymatic complex in the production of alkanes. We also obtained two members of MAH1 gene family, P450‐dependent mid‐chain alkane hydroxylase, which catalyze the hydroxylation reaction leading from alkanes to secondary alcohols and oxidation of secondary alcohols to ketones (Greer et al., 2007). However, no secondary alcohol or ketone was detected in our study. One possible reason might be that current detection condition might be not available to detect trace amount of these compounds. Besides, cuticular wax biosynthesis is controlled at the levels of transcription, some DEGs were also annotated in the “transcription” in Go and KEGG database, suggesting that light wavelengths might also be involved in the regulation of wax biosynthesis and deposition, which needs further exploration in the future.

Finally, eco‐physiological function evaluation revealed that yellow light significantly increased the leaf water loss and decreased the photosynthetica capacity (Pn, Tr and gs). Combined with the highest wax coverage under yellow light, this suggested that the changes of leaf cuticular wax might alter the leaf eco‐physiological functions. It has been shown that changes in cuticular wax amount may influence the cuticular transpiration in plants (Kim, Go, & Suh, 2018), and the removal of epicuticular wax may cause changes in the photochemical activity (Pereira, Figueiredo‐Lima, Oliveira, & Santos, 2019). In addition, the function of DEGs in faba bean leaves was also mainly related to the “plant‐pathogen interaction” and “defense mechanisms” classification (Yeats & Rose, 2013). Information on the transcriptome of faba bean leaves is essential for identifying candidate waxy genes. Further study will be required to uncover the plant defense mechanisms upon different light wavelengths in faba bean.

## 5 CONCLUSION

The regulatory mechanism of cuticular wax biosynthesis is complex and involves interacting signaling networks associated with various environmental stress responses. In this study, we observed that light wavelengths influenced the deposition of leaf cuticular wax and the expression levels of candidate genes involved in wax biosynthesis in faba bean. Light wavelengths significantly affected the contents and crystallization patterns of cuticular wax on faba bean leaves, and then posed an effect on the eco‐physiological function, such as the rate of water loss. Using Illumina platform, in total, 100,194 unigenes were obtained, and 10 genes were annotated in wax biosynthesis pathway, including VLCFAs elongation, alcohols formation, and alkane formation. The transcriptome dataset provides a theoretical basis in identifying wax functional genes of faba bean and a new way in improving crop stress resistance through genetic engineering for a rational modification of plant cuticle. Our results also prove that light at different wavelengths may also be applied in selecting candidate genes involved in wax biosynthesis in other crops.

## CONFLICT OF INTEREST

The authors declare that there is no conflict of interest.

## AUTHOR CONTRIBUTIONS

LH drafted the manuscript. YG conceived and designed the experiments. LH, XZ, XL and QX analyzed the data. LH, DW, LW, YL, ZH and LK performed the experiments. LH, QX and YG revised the manuscript. All authors have read and approved the final manuscript.

## Supporting information

Supplemental Material

Supplemental Material

Supplemental Material
