# Supplementary material for: Responses of cuticular waxes of faba bean to light wavelengths and selection of candidate genes for cuticular wax biosynthesis
Source: Plant Genome. 2020 Oct 30;13(3):e20058. doi: 10.1002/tpg2.20058 (PMC12807296; doi:10.1002/tpg2.20058)
Supplement: Supplementary file 1 [file TPG2-13-e20058-s001.pdf]

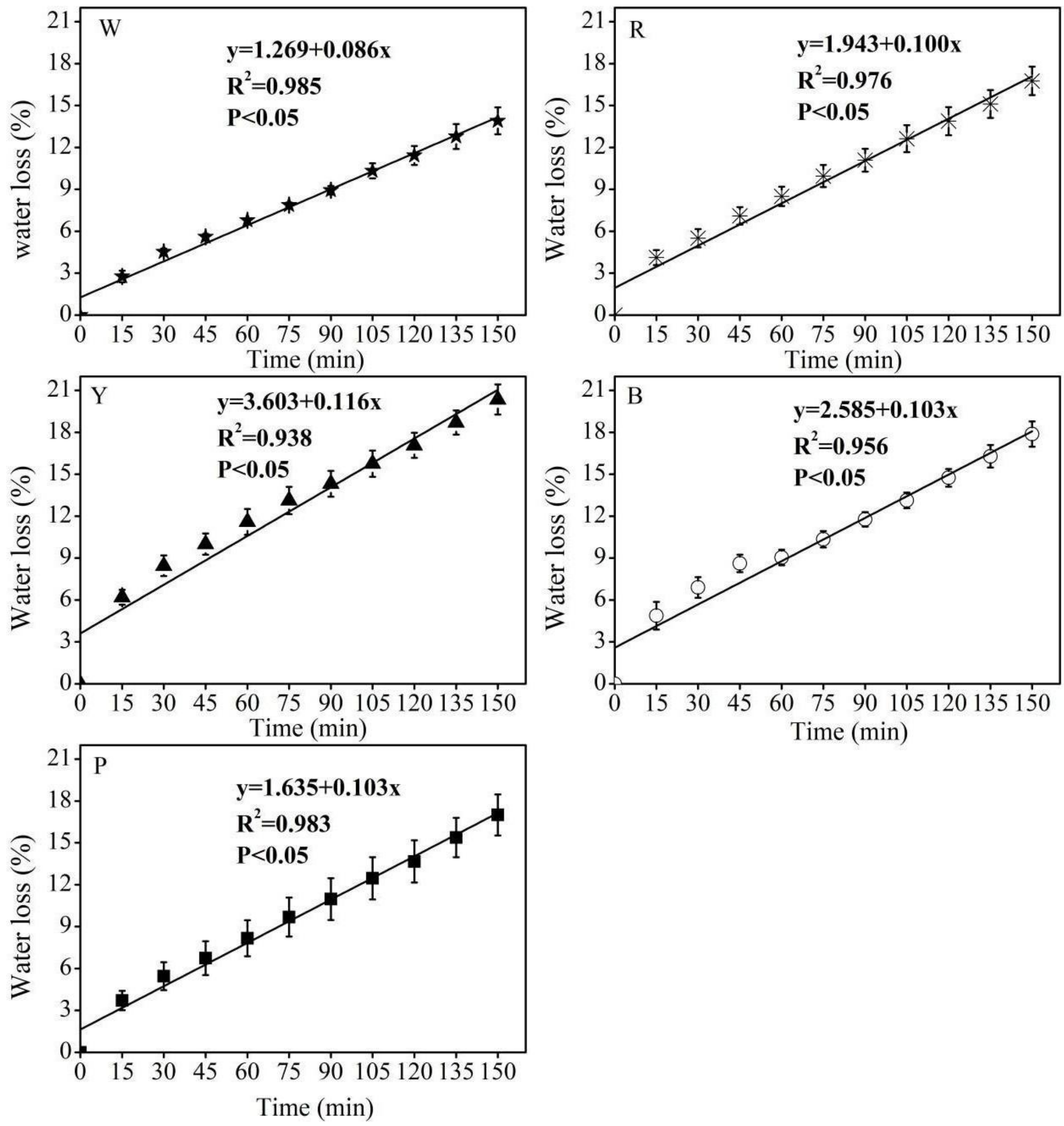


Fig S1Relationship between water loss percentage and time. W, R, Y, B and P represent white, red, yellow, blue and purple lights, respectively. The linear fitting curve was performed using OriginPro 8.0. In the equation, x stands for time, y stands for the water loss percentage, the slope stands for the rate of water loss.

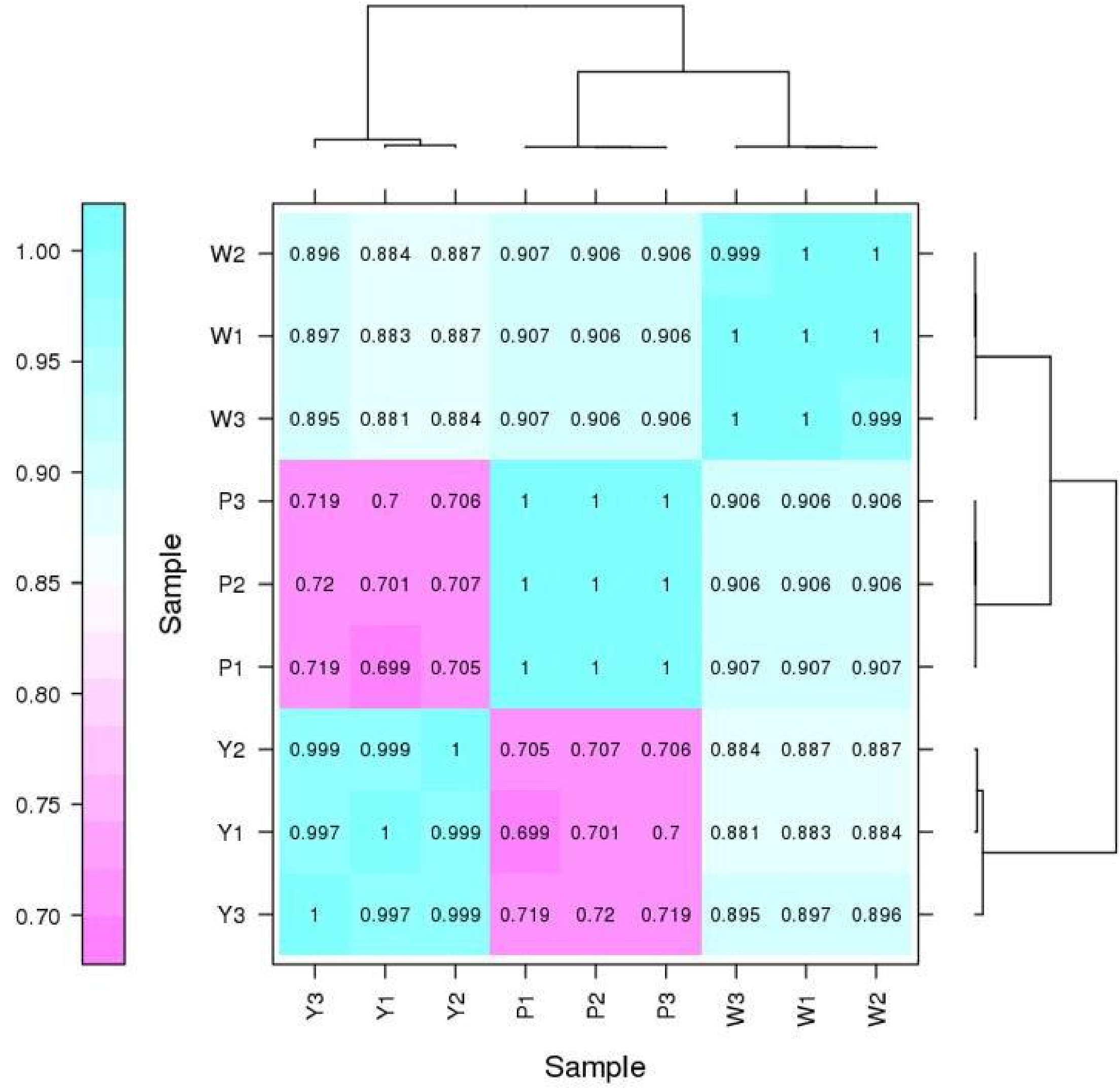


Fig S2 Correlation analysis of the expression level of genes between different samples. The left color bar is a color partition of different R2 values. R is pearson's correlation coefficient. W1, W2 and W3 represent three biological replicates from faba bean leaves under white light; Y1, Y2 and Y3 represent three biological replicates from faba bean leaves under yellow light; P1, P2 and P3 represent three biological replicates fro faba bean leaves under purple light.

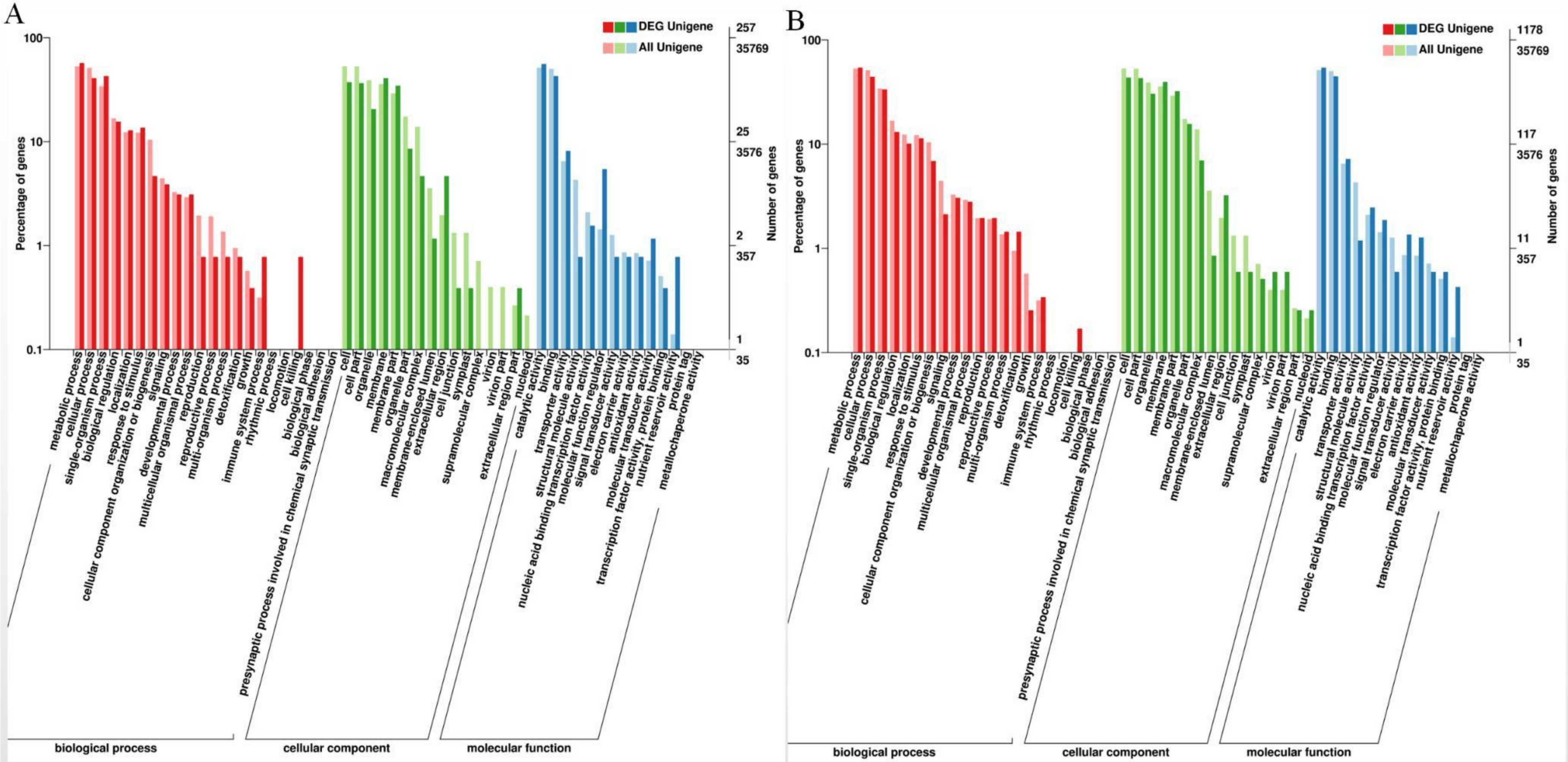


Fig S3 Histogram presentation of the Go classification of the DEGs at white vs purple lights (A) and yellow vs purple lights (B). The results were summarized in three main categories, biological process, cellular component, and molecular function. The left y-axis indicates the percentage of genes in a category, and the right y-axis indicates the number of genes in a category.

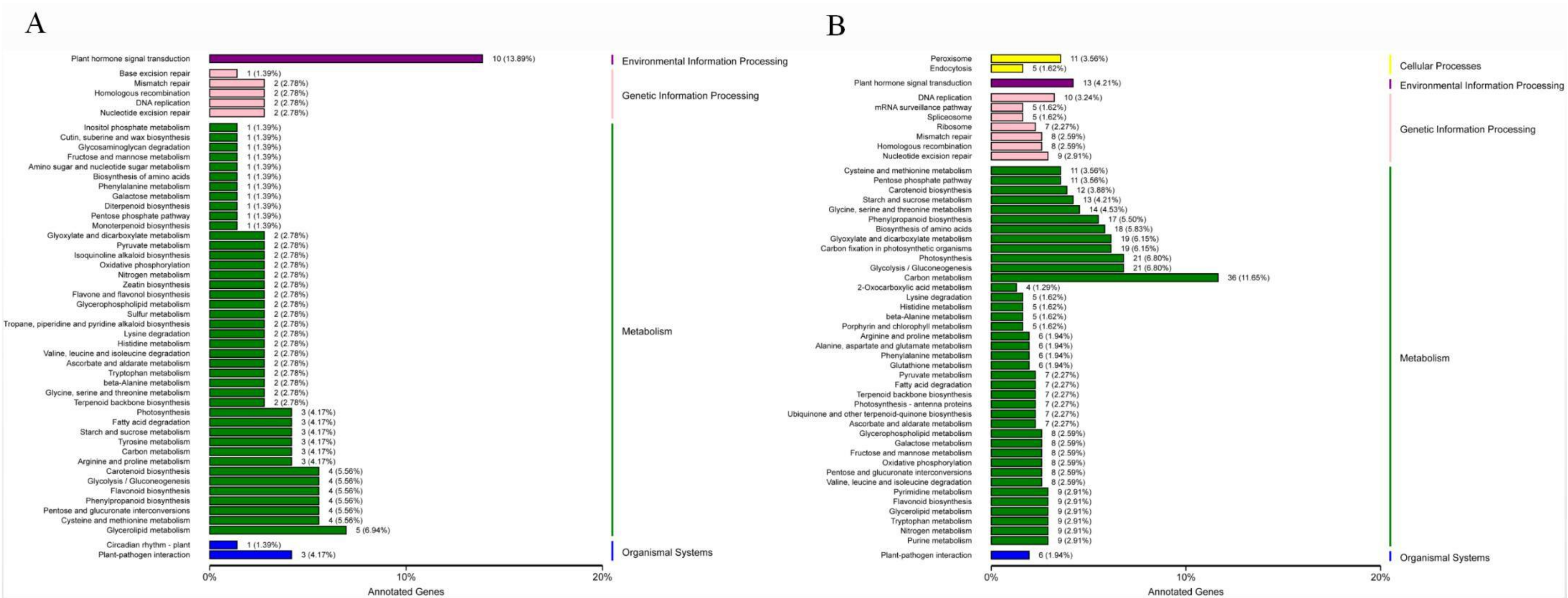


Fig S4 KEGG pathway enriched for DEGs in faba bean leaves at white vs purple lights (A) and yellow vs purple lights (B).The annotated genes was the ratio of the number of DEGs annotated in a given pathway term to the number of all genes annotated in the pathway way term. The top 50 enriched pathway items in the KEGG database were listed. Blue bar: classification based on organismal systems categories. Green bar: classification based on metabolism categories. Pink bar: classification based on the genetic information processing. Purple bar: classification based on the environmental information processing. Yellow bar:classification based on cellular processes categories.

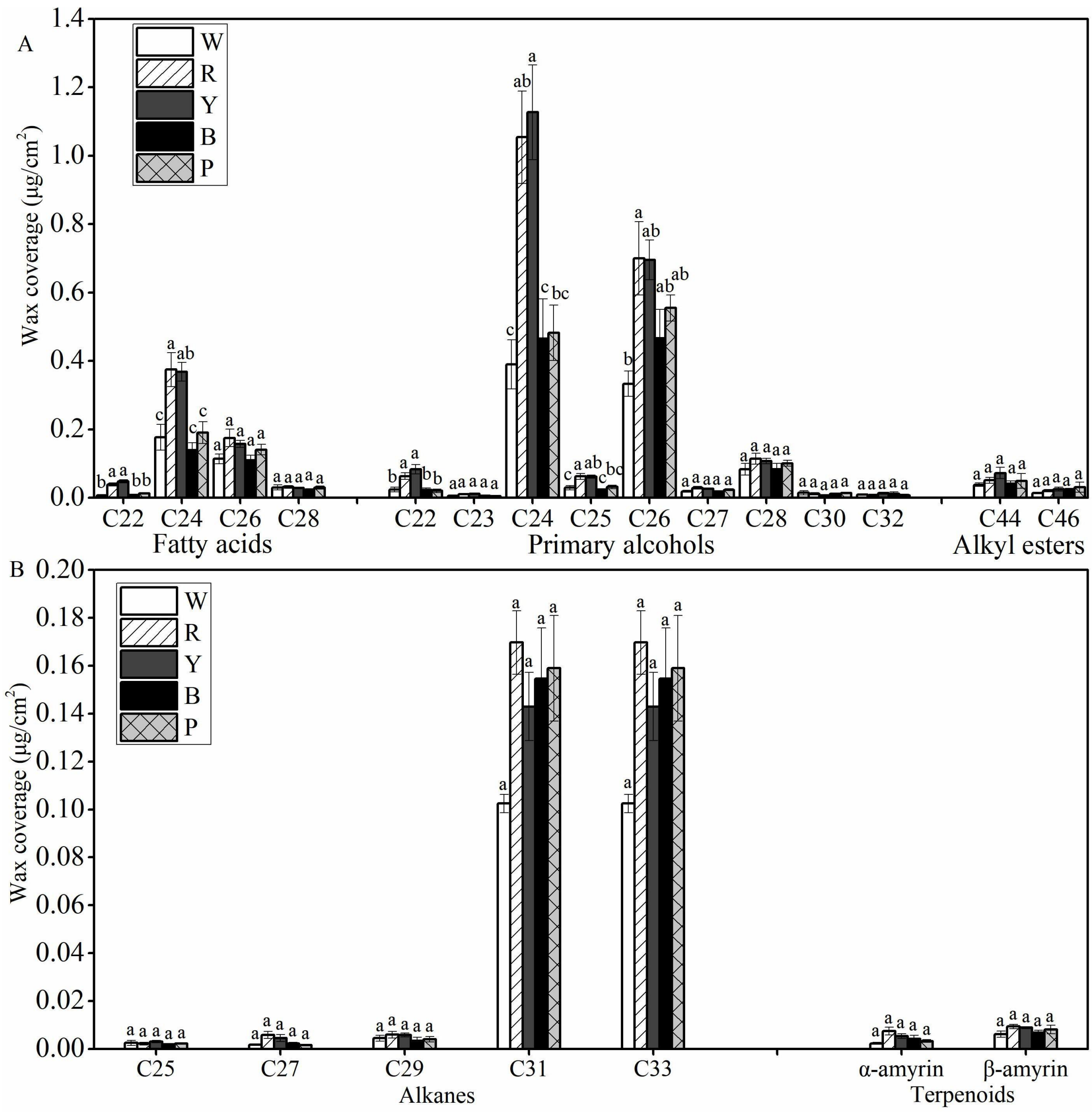


Fig S5 The contents of different wax component with varied chain lengths in leaves of faba bean grown under different light treatments. (A) The contents of fatty acids, primary alcohols and alkyl esters. (B) The contents of alkanes and terpenoids. W, R, Y, B and P represent white, red, yellow, blue and purple light, respectively. The error bar represents standard error. Different lowercase letters above data bars within each compound class represent significance at *P<0.05* according to the least significant difference test.
