# Supplementary material for: Responses of cuticular waxes of faba bean to light wavelengths and selection of candidate genes for cuticular wax biosynthesis
Source: Plant Genome. 2020 Oct 30;13(3):e20058. doi: 10.1002/tpg2.20058 (PMC12807296; doi:10.1002/tpg2.20058)
Supplement: Supplementary file 2 [file TPG2-13-e20058-s002.pdf]

Table S1 Effects of different lights on the ACL and CPI of wax compounds

| Light | ACL | | | CPI |
|---|---|---|---|---|
| | Fatty acid | Primary alcohol | Alkane | Primary alcohol |
| W | 25.04 | 25.33 | 31.41 | 0.07 |
| R | 24.64 | 24.97 | 31.42 | 0.05 |
| Y | 24.56 | 24.92 | 31.42 | 0.05 |
| B | 25.06 | 25.34 | 31.53 | 0.05 |
| P | 25.02 | 25.40 | 31.54 | 0.05 |

Note: W, R, Y, B and P represent white, red, yellow, blue and purple light, respectively.

Table S2 Functional annotation of the unigenes from faba bean

| Annotated Database | Annotated_Number | 300≤length<1000 | length≥1000 |
|---|---|---|---|
| COG | 16963(16.93%) | 6710 | 6045 |
| GO | 35769(35.70%) | 15342 | 11574 |
| KEGG | 21209(21.17%) | 9599 | 6197 |
| KOG | 32914(32.85%) | 14317 | 9934 |
| Pfam | 37707(37.63%) | 14596 | 15917 |
| Swissprot | 32852(32.79%) | 14098 | 11656 |
| eggNOG | 54500(54.39%) | 23516 | 15271 |
| NR | 51683(51.59%) | 22755 | 15893 |
| All | 60614 | 26613 | 16147 |

Table S3 Primers of selected genes used for qRT-PCR analysis

| Unigene | Primer sequence (5'-3') | Product length (bp) | Tm (℃) |
|---|---|---|---|
| >c49821.graph_c0,c1,c3,c4 | F:TTACACTGTGAAGGTTGAGGAAGG<br>R:AGGGGAGTCTAAACCAGAAGGC | 127 | 61.4 |
| >c44810.graph_c0 | F:AATGGAAACGAGTAGCGACG<br>R:CAACTGTCACATTTCGCCTG | 218 | 61.4 |
| >c54135.graph_c0 | F:AAATCTATGTATGGAAGAAGCCCG<br>R:TCAACCCAGCACTACAGCCC | 233 | 61.4 |
| >c57811.graph_c0 | F:GACGATTACCCGATTTTCTCC<br>R:GGAGCAAGTAATGACACAGACG | 203 | 57 |
| >c56940.graph_c1 | F:TTCATTCGCTACCTTCTCCCA<br>R:CCAACCGCAACTTTTCTGTG | 189 | 57 |
| >c58237.graph_c0 | F:TCAAGACTGAAATGAAAACGACC<br>R:TTACCCCATTTGGCTTTTCC | 246 | 63.3 |
| >c53753.graph_c1 | F:AAAAAAGGCACCAAAAGGAGC<br>R:GGACATTGGCTAAAGAAGGAGG | 125 | 61.4 |
| >c59464.graph_c1 | F:AGATGTGGAAGGTCTCGGTTG<br>R:ACACGTTGTGACTGGTTTTAAGC | 190 | 61.4 |
| >c57653.graph_c0 | F:AAGTGTGCAGTCAAATCTGATGTAC<br>R:AATGCTATGAACTTGTAAGATGGC | 194 | 61.4 |
| >c60650.graph_c2 | F:CTGGCTATGGCTATCACACTCA<br>R:CAAGGTCCTAAGAACTCAACTGTG | 200 | 61.4 |
| 18 S | F:TCAAGGTCGGGGACAACAGT<br>R:ACGGACTCTTTACGCCCAAT | 394 | 61.4 |
